# Complex Left Main Trifurcation: A Case Study of Successful Treatment

**DOI:** 10.3390/jcm14020328

**Published:** 2025-01-08

**Authors:** Marius Rus, Mihnea-Traian Nichita-Brendea, Mircea-Ioachim Popescu, Georgeta Pașca, Claudia Elena Staniș, Simina Crișan

**Affiliations:** 1 Department of Medical Disciplines, Faculty of Medicine and Pharmacy, University of Oradea, 410073 Oradea, Romania; 2 Cardiology Department, Bihor Clinical Emergency Hospital, 410169 Oradea, Romania; 3 Department of Psycho Neuroscience and Recovery, Faculty of Medicine and Pharmacy, University of Oradea, 410073 Oradea, Romania; 4 Faculty of Medicine and Pharmacy, University of Oradea, 410073 Oradea, Romania; 5 Cardiology Department, “Victor Babes” University of Medicine and Pharmacy, 2 Eftimie Murgu Sq., 300041 Timisoara, Romania

**Keywords:** acute myocardial infarction, percutaneous coronary intervention, triple-vessel coronary artery disease, left main trifurcation, Triple-Kissing Balloon, multi-stent implantation

## Abstract

**Objectives:** True trifurcation disease of the left main coronary artery is a rare situation encountered in clinical practice. To date, there is no evidence for a standardized strategy of percutaneous coronary intervention in this type of lesion. **Methods:** This article describes a novel three-stent strategy using a combination of Triple-Kissing Balloon Crush in both of the side branches. This technique is based on a well-established bifurcation stenting technique, namely, the Double-Kissing Crush technique. **Results:** This strategy was implemented successfully, demonstrating technical feasibility and optimal stent apposition in the trifurcation lesion, ensuring the preservation of all three branches. **Conclusions:** Although more data and clinical trials are needed to develop proper evidence-based guidelines, three-stent implantation with Double-Trissing Crush should be taken into consideration as a viable strategy for LM trifurcation lesions in the proper set of patients.

## 1. Introduction

The first left main percutaneous coronary intervention (PCI) was performed by using plain old balloon angioplasty (POBA). In his initial experience, Gruntzig performed POBA in 50 patients over a period of 18 months, deeming it successful in 32 patients [1]. After decades of technical improvement and new techniques development, PCI has emerged as a valuable alternative to coronary artery bypass grafting (CABG) in the management of left main coronary artery (LM) disease [2]. If LM bifurcation (LMB) has become a fairly common occurrence in the catheterization laboratory, and its management no longer represents a herculean task with the help of advanced techniques such as provisional stenting (PS), Double-Kissing Crush (DK-Crush), Culotte, or T-and-small-protrusion (TAP), LM trifurcation sets an interesting challenge for interventional cardiologists.

LM trifurcation (LMT) represents the division of the main stem of the left coronary artery into three branches instead of two [3]. Two main classifications have been proposed for LMT disease: the modified Medina classification and the classification proposed by Shammas et al. [4,5]. Current guidelines recommend that only patients with LM disease and a low “Synergy Between PCI With Taxus and Cardiac Surgery” (SYNTAX) score (below 22) should be treated with PCI as a class I recommendation [6,7]. The SYNTAX trial 5-year results reported a higher number of major adverse cardiac events (MACE) among the high-SYNTAX score group of patients [8]. The EXCEL trial (“Evaluation of XIENCE Versus Coronary Artery Bypass Surgery for Effectiveness of Left Main Revascularization”) further supports these findings [9].

The DKCRUSH-V Trial notes an advantage of the DK-Crush technique over PS, regarding target lesion failure and stent thrombosis for LMB disease [10].

Comparing LMT and LMB, the EXCEL trial notes no difference in MACE in the 5-year follow-up period, the LMT group displaying less complex atherosclerosis involving LM [11]. Due to the rarity of LMT procedures, most studies reporting the outcomes of LMT patients undergoing PCI included a small number of cases, with the Milan–New Tokyo registry being the largest one to date [11].

LMT represents a unique anatomical challenge for interventional cardiologists, requiring a thorough understanding of its classification, anatomy, and potential outcomes. A multidisciplinary approach combining the expertise of interventional cardiologists, cardiovascular surgeons, and researchers is crucial in understanding the optimal management of LMT disease in order to develop adequate evidence-based guidelines for its treatment and thus deliver the best patient care. We present herein a case of true LMT where an adaptation of the DK-Crush technique, albeit on three branches, was carried out.

## 2. Case Presentation

A 63-year-old male patient presented to our institution with the clinical and paraclinical characteristics of an acute inferior ST-elevation myocardial infarction (STEMI). Urgent coronary angiography was performed revealing multi-coronary artery disease (MVD) involving a true trifurcation of the LMCA lesion (Medina 0.1.1.1). The culprit lesion in the right coronary artery was treated by primary percutaneous coronary angioplasty (PPCI).

The patient presented multiple cardiovascular (CV) risk factors (hypertension, former smoker), with no history of CV disease. He addressed the emergency department (ED) for acute constrictive chest pain for 6 h prior to the presentation. The initial ECG revealed 2–3 mm ST-elevation in the inferior leads (DII, DIII, aVF) and the right leads (V3R, V4R), with reciprocal ST-depression in the lateral leads (DI, aVL). The level of high-sensitive troponin T was significantly increased. The transthoracic echocardiography revealed a 57% ejection fraction of the left ventricle (LVEF), hypokinesia of the posterior wall, and inferior interventricular septum, with a non-dilated and contractile right ventricle. The diagnosis of acute inferior STEMI was established, and the patient underwent coronary angiography. Dual anti-platelet therapy (acetylsalicylic acid and ticagrelor) was initiated, and the patient was transferred to the cardiac catheterization laboratory in the first hour after admission.

**Figure 1:**
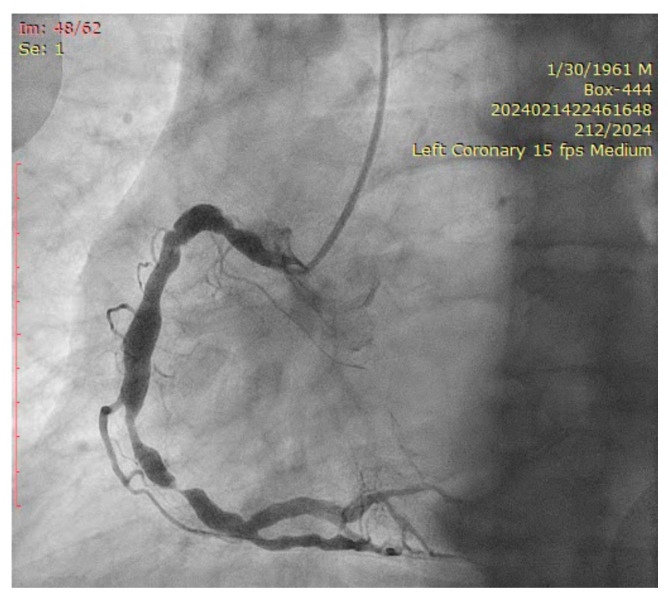
Right coronary artery (Left anterior oblique view—LAO).

**Figure 2:**
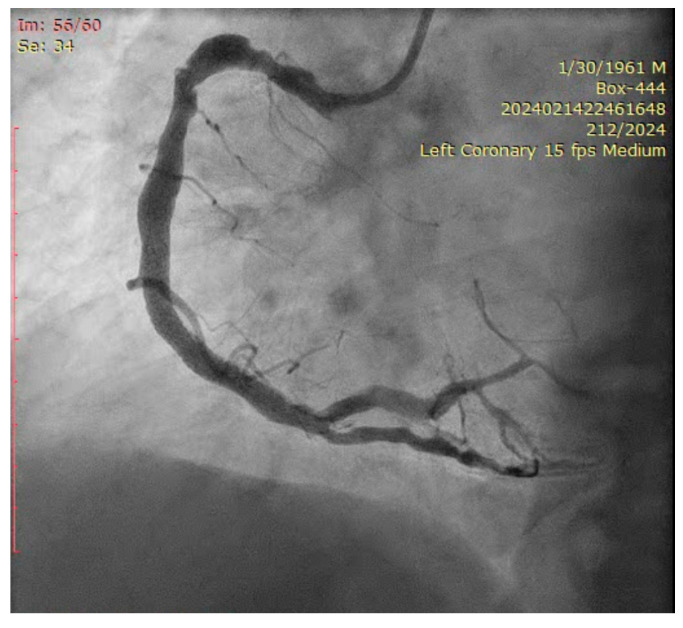
RCA result post-procedure.

The coronary angiogram revealed multi-vessel coronary artery disease (MVCAD), with the right coronary artery(RCA) revealed as the culprit vessel (Figure 1). The diagnostic procedure was followed by successful PPCI (Figure 2), with the implantation of three stents (5.0/12, 5.0/12 and 4.5/12 mm Everolimus-eluting stents—Synergy, Boston Scientific) in the RCA and optimal angiographic, procedural, and clinical results.

**Figure 3:**
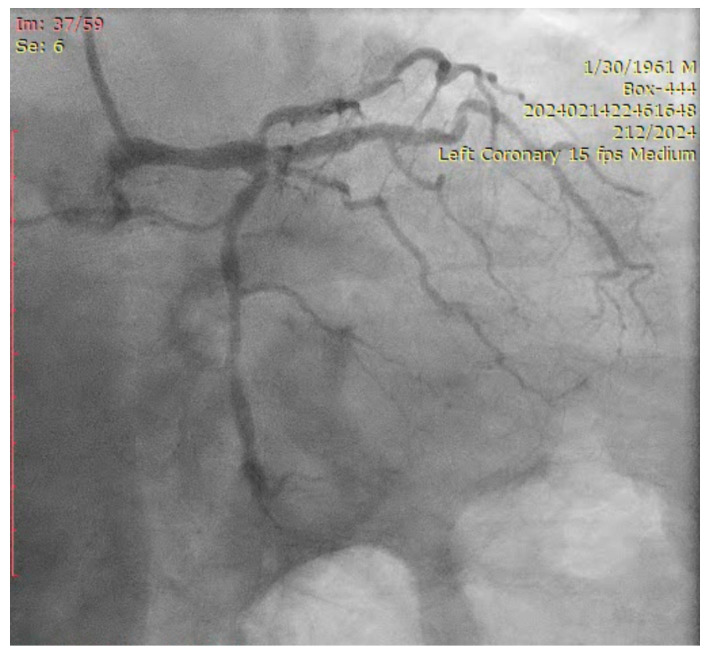
Left coronary artery (Right anterior oblique caudal (RAO) caudal view).

**Figure 4:**
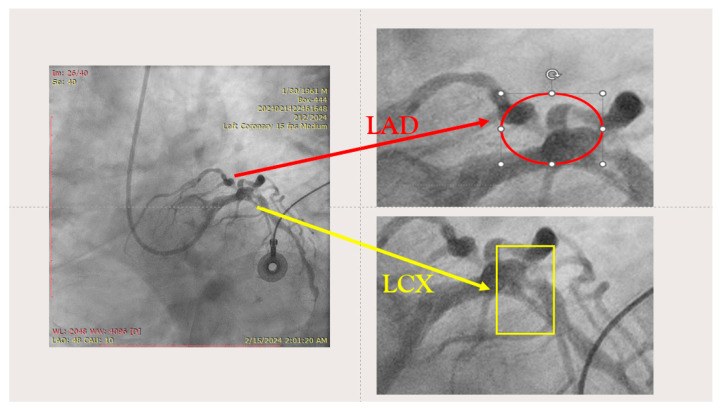
Left coronary artery—LAD and LCX (LAO caudal). Red arrow and circle—ostium of LAD. Yellow arrow and square—ostium of LCX.

**Figure 5:**
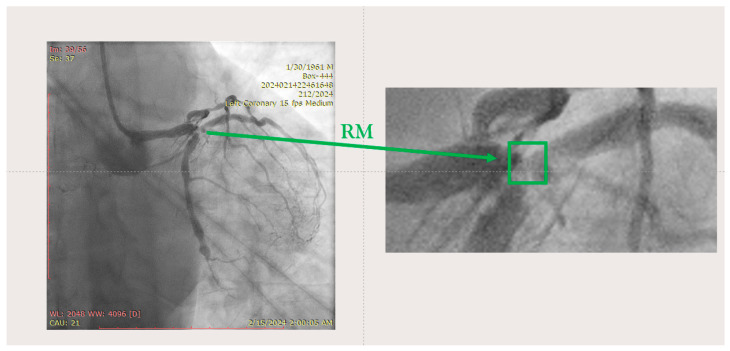
Left coronary artery—RM(LAO caudal). Green arrow and square—ostium of RM.

**Figure 6:**
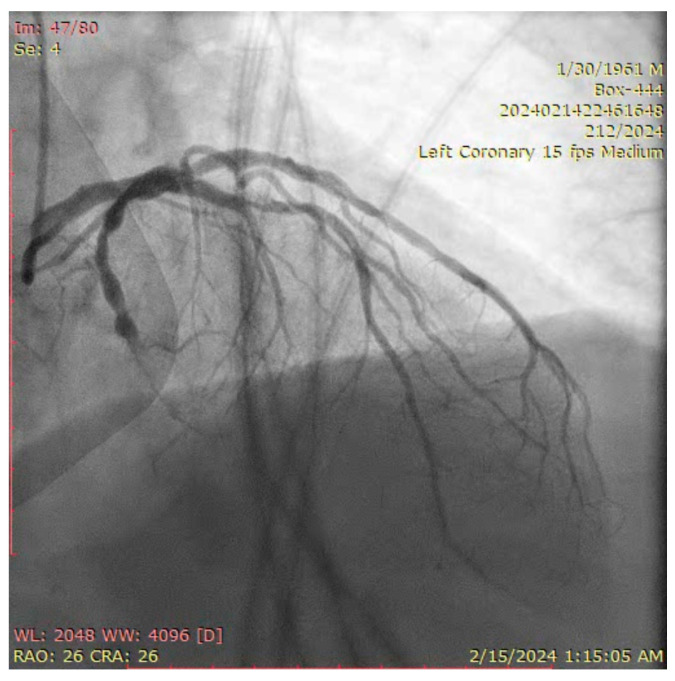
Left coronary artery (RAO cranial).

**Figure 7:**
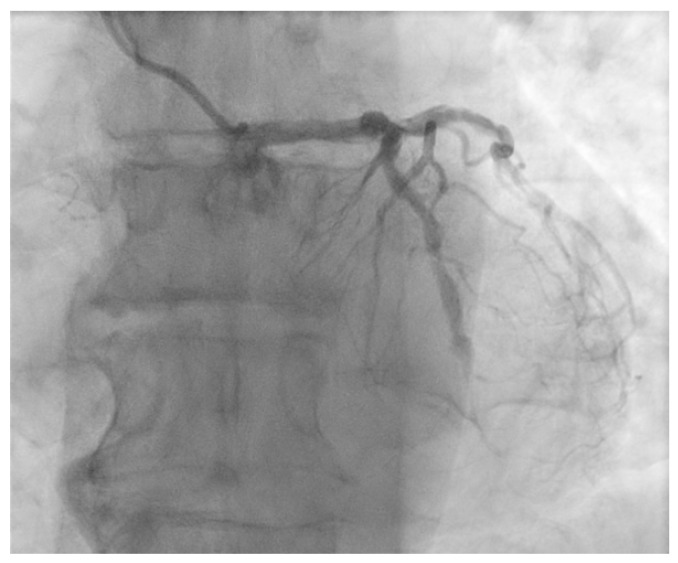
Left coronary artery (LAO cranial).

**Figure 8:**
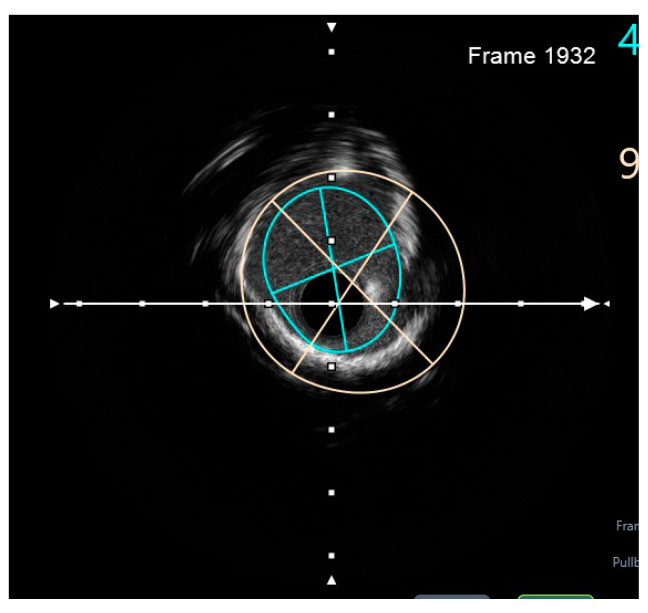
Preprocedural IVUS LCX. White arrow—axis.Orange circle—vessel area. Blue circle—remaining luminal area.

**Figure 9:**
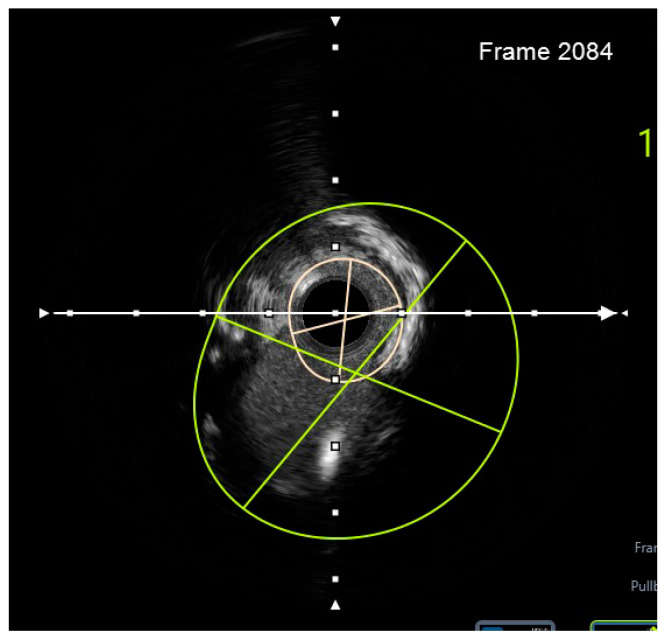
Preprocedural IVUS LAD. White arrow—axis. Yellow circle—vessel area. Gray circle—remaining luminal area.

**Figure 10:**
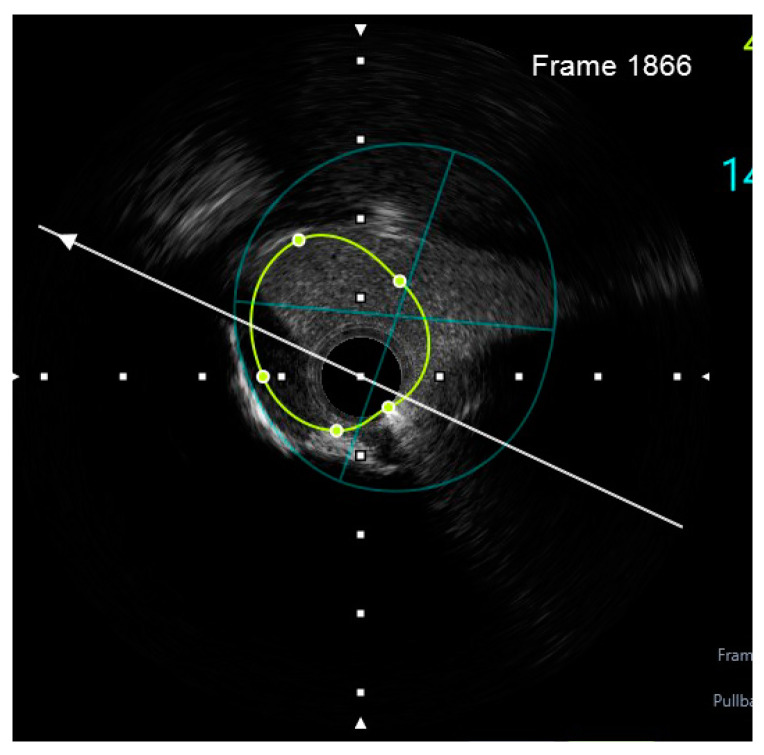
Preprocedural IVUS RI. White arrow—axis. Yellow circle—vessel area. Blue circle—remaining luminal area.

The ostium of left anterior descending artery (LAD) presented a 75% stenotic lesion, with the proximal segment displaying a 90% lesion (Figure 3). The left circumflex artery (LCX) revealed a 75% stenotic lesion of the ostium and tapered disease of its distal segment with areas of ectasic disease, with the ostium of the ramus intermedius (RI/RM) showing a 75% lesion (modified Medina classification 0-1-1-1 [4]) (Figure 4, Figure 5, Figure 6, Figure 7, Figure 8, Figure 9 and Figure 10).

The SYNTAX score was calculated at 34, classifying this case as an extensive angiographic lesion. The calcium score calculated through intravascular ultrasound (IVUS), as well as the tortuosity of the vessels, further added to the complexity of the lesion. The EuroSCORE II presented a 1.62% mortality rate at 30 days post-CABG, a higher risk compared to the British Columbia PCI score of 1.1% for 30-day mortality rate following PCI. According to the current revascularization and STEMI guidelines (both the ESC and ACC/AHA), MVCAD patients who undergo complete revascularization present better short-and long-term outcomes [6,7].

Taking into consideration the current guidelines recommending complete coronary revascularization, as well as the high SYNTAX score, the case was discussed within the Heart Team, and a surgical solution was proposed to the patient. Nonetheless, the patient refused CABG, opting instead for PCI. According to the consensus of the EBC (European Bifurcation Club), a “one-stent strategy” when possible is more favourable than a multiple stent strategy [12]. However, a two-stent strategy is sometimes favoured by the vessel anatomy, with the DK-Crush technique proving itself to be the most advantageous [10]. The presence of a true trifurcation raises concerns about the feasibility, the technical aspects of a two-stent strategy, as well as the need of a Trissing technique. If an additional stent is needed, the complexity becomes even higher.

Due to the patient’s refusal to undergo CABG, we opted to complete the revascularization by PCI at 30-day interval post-procedure for the culprit vessel. Taking into consideration the complex anatomical challenge that a true Medina 0.1.1.1 trifurcation lesion poses and the need to preserve access to both side-branches, as well as the distal main branch (MB), we opted for a three-stent strategy similar to the DK-Crush technique used in LMB. We adapted this technique to this particular anatomical setting, namely Double-Trissing instead of Double-Kissing.

**Figure 11:**
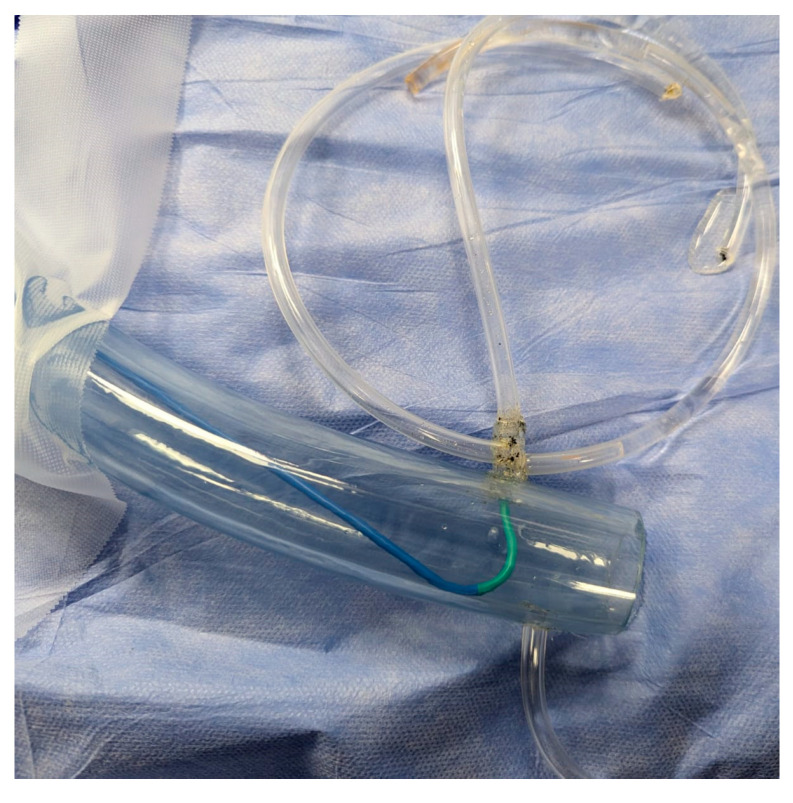
In vitro procedure.

**Figure 12:**
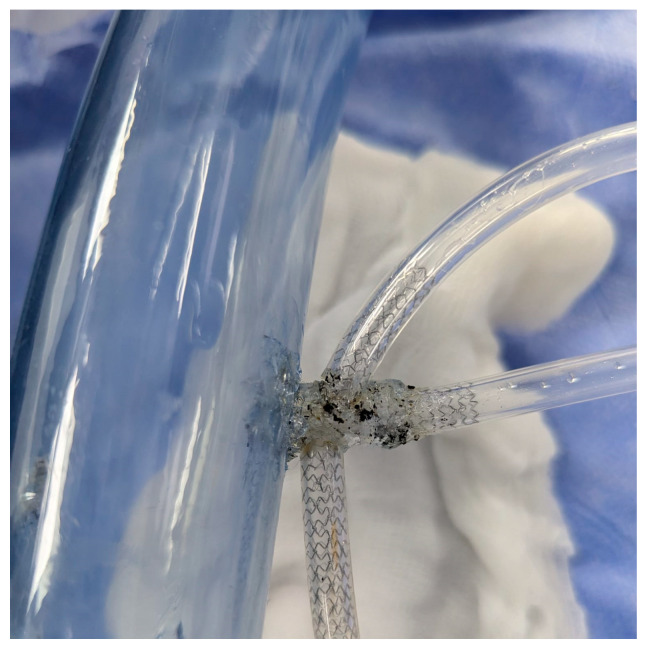
In vitro procedure—model close-up.

**Figure 13:**
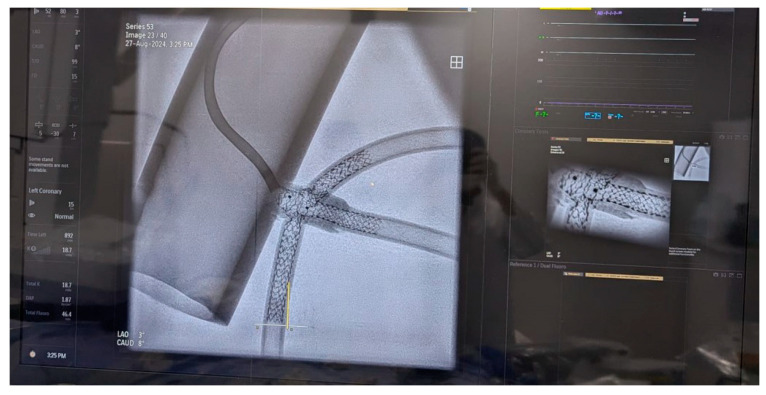
In vitro procedureunder X-rays.

In preparation for the in vivo intervention, we planned the steps and performed an in vitro procedure to see what happens to the crushed stents, the amount of overlay stent struts at the carinas level, and the main vessel stent deformation related to the post-dilation (Figure 11, Figure 12 and Figure 13).

As the in vitro procedure displayed promising results and our team discussed and made preparations for every possible scenario, we went ahead with implementation of the technique in our patient after properly presenting them with the risks and benefits of the new intervention.

**Figure 14:**
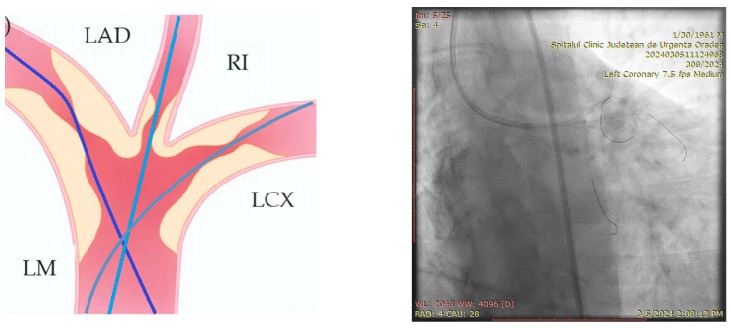
Step 1.

**Figure 15:**
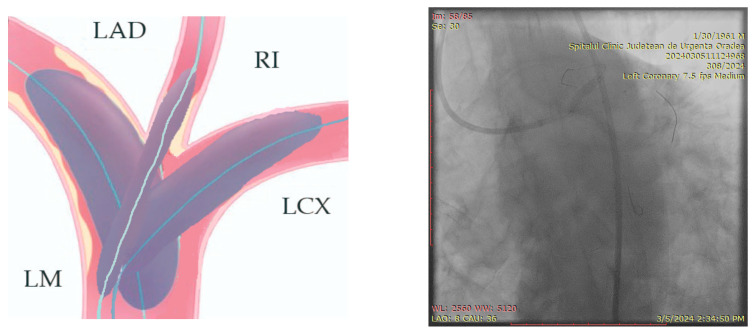
Step 2.

**Figure 16:**
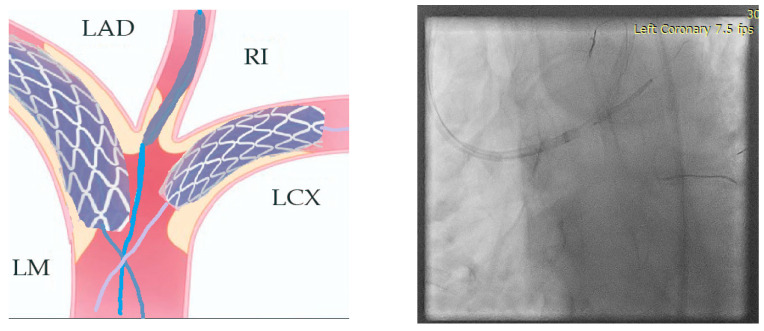
Step 3.

**Figure 17:**
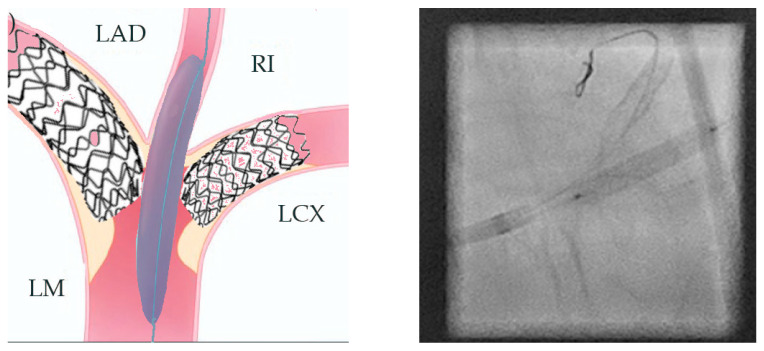
Step 4.

**Figure 18:**
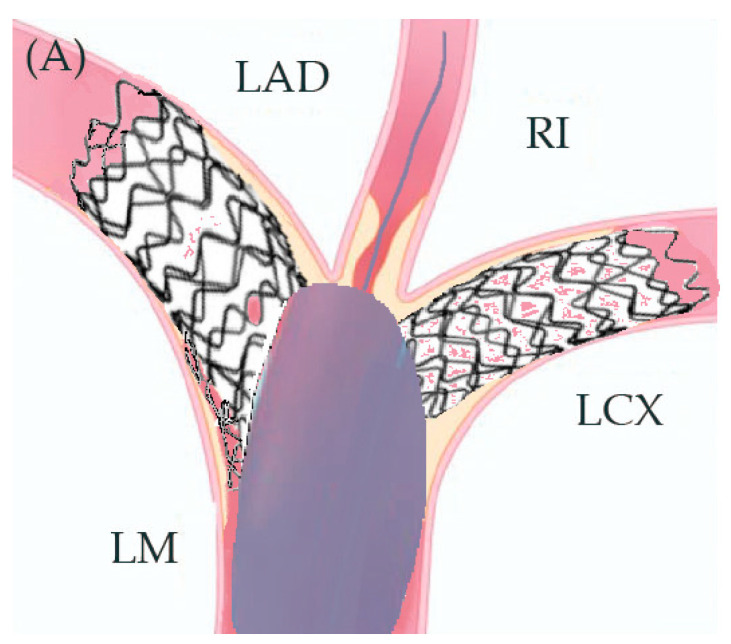
Step 5.

**Figure 19:**
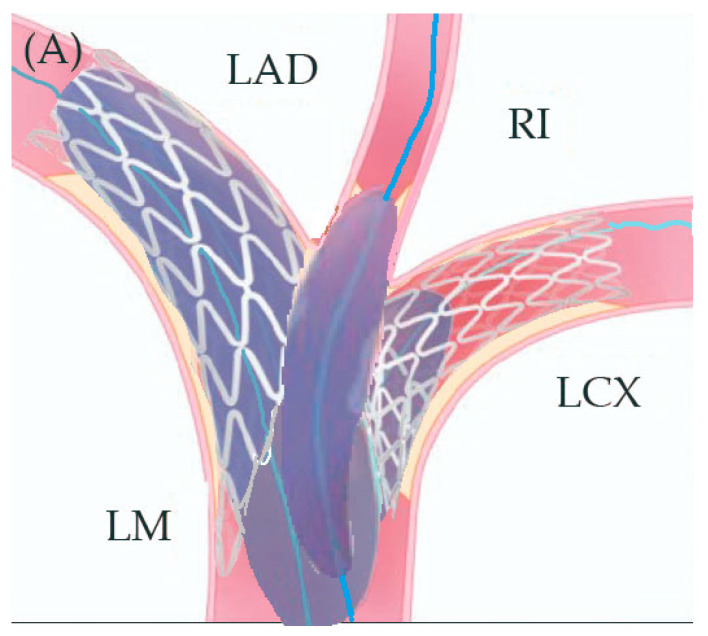
Step 7.

**Figure 20:**
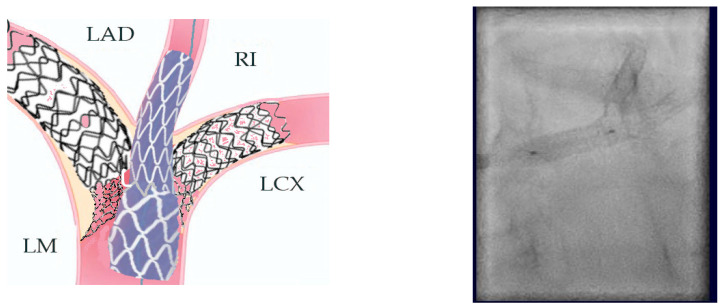
Step 9.

**Figure 21:**
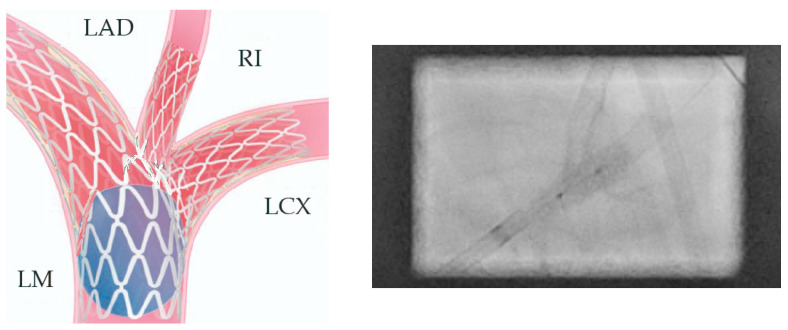
Step 10.

**Figure 22:**
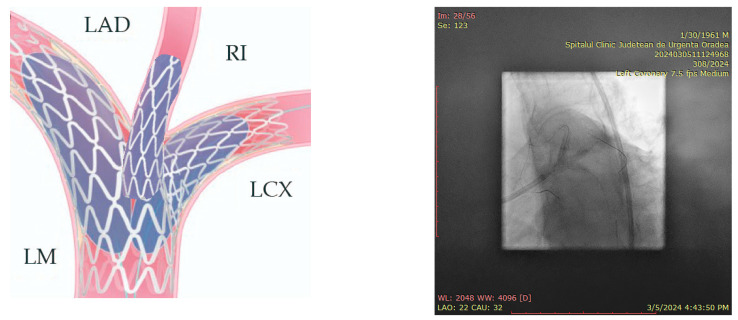
Step 12.

**Figure 23:**
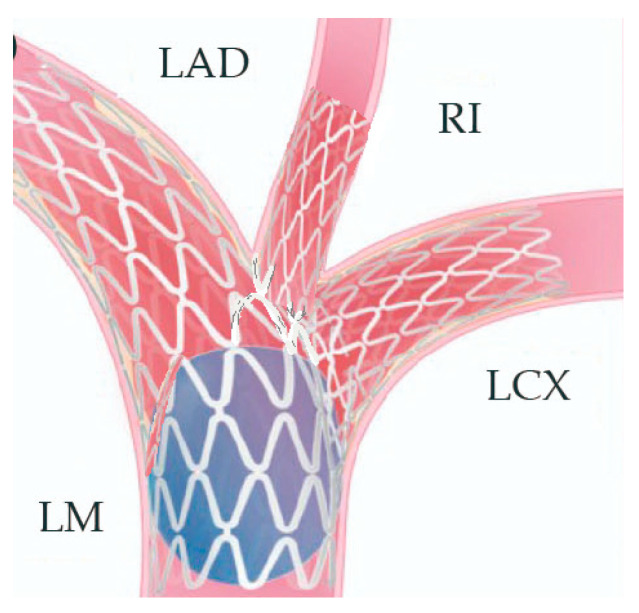
Step 13.

**Figure 24:**
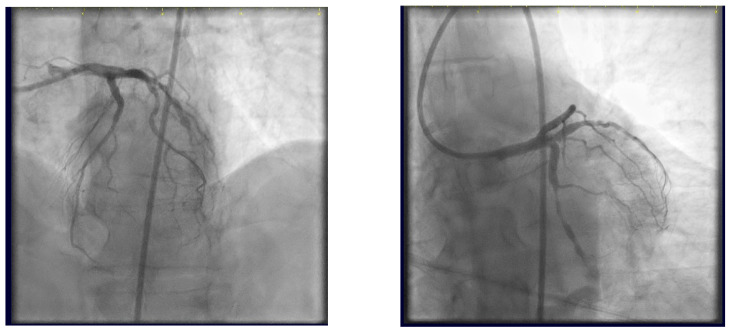
Final result.

**Figure 25:**
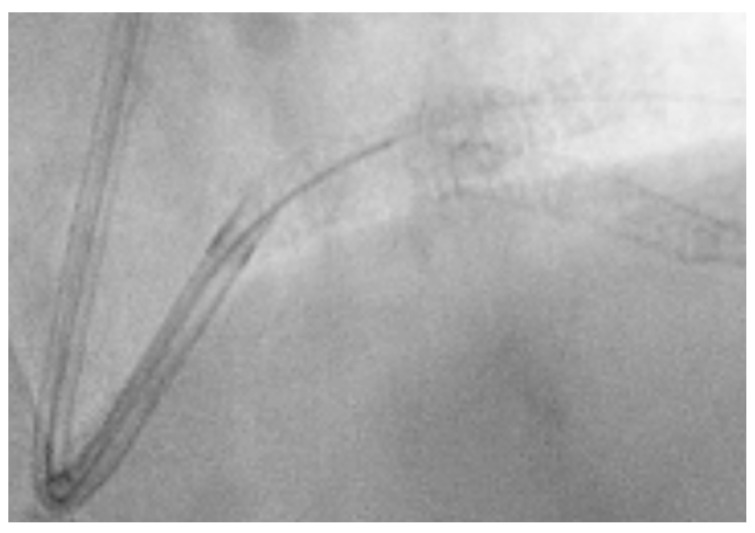
Final result, LM coverage.

The Double Triple-Kissing (Trissing) Crush is a novel technique consisting of the following steps:

1. Engage the left coronary artery with an EBU 4.0/8F guiding catheter. Wiring the three branches of the trifurcation (Floppy guide wires in LAD and RI, Hydrophilic guide wire in LCX) (Figure 14).

2. Performing the first Trissing with 1:1 NC balloons sized to the distal branches’ diameter (3.5, 3.5, 4.0 mm) (Figure 15).

3. Stenting the two most angled branches with stents (3.5/28 mm and 3.5/19 mm Sirolimus-eluting stents—Ultimaster, Terumo) sized to their distal diameter and slight protrusion in the main branch (Figure 16).

4. The two stents implanted in the side branches should be crushed with a balloon placed in the MB and sized 1:1 to the distal diameter of the vessel (3.5/18 mm). Remove the side-branch guidewires (Figure 17).

5. First POT (proximal optimization therapy) with a 1:1 balloon sized (5.5) to the proximal part of the MB (Figure 18).

6. Recrossing with the guidewires in the side branches and a small pre dilatation of the crushed struts with a small 1.2–1.5 balloon in order to accommodate the balloons for the Trissing.
7. Second Trissing (balloon NC 3.5/18 mm) (Figure 19).

8. An additional POT is performed at this stage.
9. A 1:1 stent sized to the distal main vessel diameter is placed from the proximal to the distal main branch (5.0/12 mm Everolimus-eluting stent—Synergy, Boston Scientific) (Figure 20).

10. POT is performed in order to appose the struts of the main vessel stent to the vessel wall in the proximal segment (Figure 21).

11. The crossing of the side branches is performed with guidewires, and a small dilatation of the struts with 1.2–1.5 balloons is performed.
12. The third and final Trissing is performed (3.5/20 mm, 3.5/20 mm, and 4.0/20 mm) (Figure 22).

13. Final POT is the last step of the procedure (5.5 balloon) (Figure 23).Figure 24 and Figure 25 show the final result.

**Figure 26:**
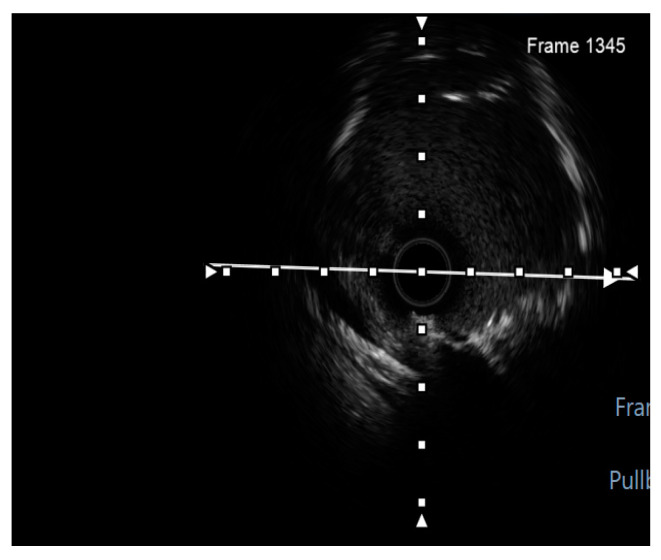
RM pullback-length of three strut layers 4 mm. White arrow—axis.

**Figure 27:**
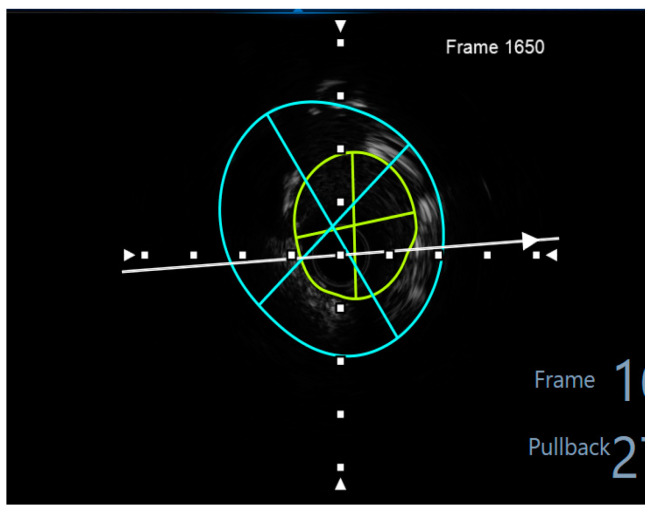
The minimum stent area at the level of the LCX4.37 mm^2^. White arrow—axis. Blue circle—vessel area. Yellow circle—remaining luminal area.

**Figure 28:**
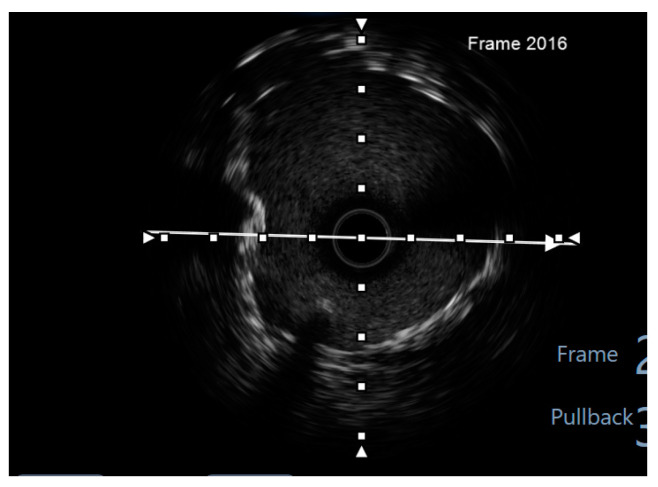
LCX pullback. Polygon of confluence. White arrow—axis.

**Figure 29:**
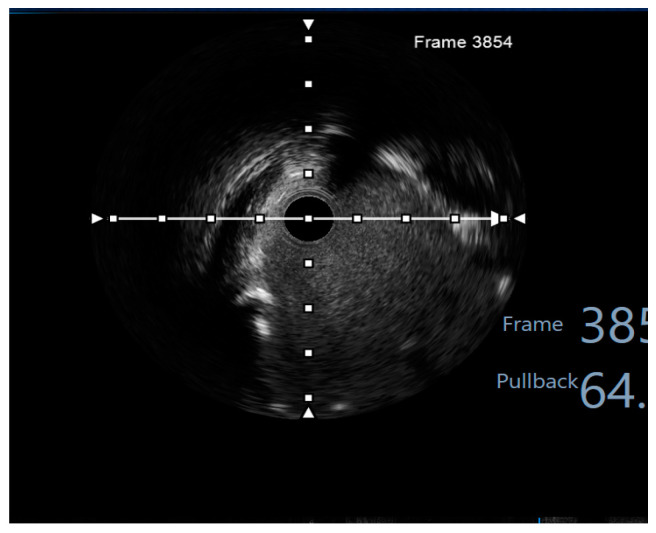
LAD pullback. Polygon of confluence. White arrow—axis.

Finally, intravascular ultrasound (IVUS) revealed adequate expansion with an optimal angiographic result and no periprocedural complications (Figure 26, Figure 27, Figure 28 and Figure 29).

**Figure 30:**
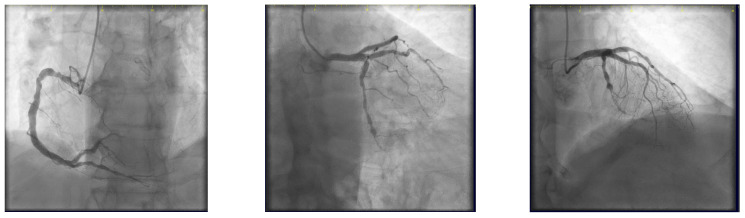
Control angiography at 6-month follow-up.

At the 6-month follow-up, the control angiography reveals patent stents at the trifurcation level (Figure 30).

## 3. Discussions

This article presents a first-in-man proof of concept for a three-stent strategy employed in a true Medina 0.1.1.1 trifurcation, modelled after the DK-Crush technique (used in LMB), in which the Two-Kissing steps are replaced by Two-Trissing steps. While the technique ensures the preservation of all three branches of the LM, the following significant limitation persists: the middle branch, specifically the ramus intermedius artery, must always serve as the technical main branch, and needs to support a minimum 3.5 mm stent platform. Additionally, the procedure’s high complexity, involving numerous steps, further reduces its practicality and reproducibility.

Considering the complexity of LMT lesions, careful planning of the strategy, as well as the materials needed, is crucial for the most favourable outcome [13]. Despite the several techniques available, the optimal strategy for left main trifurcation lesions remains uncertain [14].Some studies might argue that the main difference between true LM bifurcations and trifurcations is simply the need to protect two SBs instead of one [13]. This might require inflating three balloons at the same time, a technique displaying excellent results [13,15]. Thus, a large 7 or 8F guide is recommended, despite the higher risk of significant blood loss. In the presented case, to properly employ all the needed materials, we also used an 8F guiding catheter.

To date, there are limited data comparing one- and two-stent techniques for the approach of LMB lesions, and no data regarding three-stent approaches. The DKCRUSH-III study highlighted the increased MACE rate at the 3-year post-PCI landmark of the Culotte stenting techniques, compared to DK-Crush [16]. The DKCRUSH-V Trial compared DK-Crush with PS, with the PS increasing the risk of target lesion failure and stent thrombosis [10]. The COBIS (Coronary Bifurcation Stenting) II registry also displayed an increased incidence of target lesion failure (TLF), cardiac death, and myocardial infarction (MI), after double-stenting maneuvers for LMB cases [17]. On the other hand, the DEFINITION study revealed favourable clinical outcomes using two stents in complex LMB cases, compared to one-stent strategies [14]. More recently, the European bifurcation club Left Main Coronary Stent (EBC MAIN) study found no statistically significant difference in MACE comparing PS approach to planned dual stenting [18].

Considering the large area served by the SBs, the high plaque burden at the carina, and the need for SB bailout, double- or triple-stent implantation is needed in some cases, such as our case [19]. Kuboet al. evaluated the safety and feasibility of DESs implantation with Triple-Kissing-balloon technique (KBT, also called Trissing/menage a trois) for LM trifurcation lesions, compared to single-stent procedures [19]. The results were favourable clinically and angiographically for multi-stent Triple-KBT, while the single-stent procedures exhibited lower target lesions revascularization (TLR) rate at the 3-year follow-up, but no TLR for both groups beyond the first year post-PCI [20]. On the other hand, the Milan–New Tokyo registry reported an increased rate of in-stent restenosis for multi-stent procedures compared to the single-stent strategy, often involving ostial LCX [2].

Multiple attempts to tackle trifurcation lesions have been reported over the years, such as the “W” stenting, or procedures combining DK-Crush with PS or Culotte technique. One study has presented a successful multi-stent implantation strategy for LMT lesions, even in acute settings, with favourable clinical outcomes [20]. At the same time, Hernandes-Floreset al. report an efficient technique using dual-guide catheters and a complete radial approach [21]. An interesting technique for LMT lesions was also proposed using simultaneous Jailed Balloon and Jailed Corsair Technique [22], while a sequential strategy combining DK-Crush and Jailed balloon technique for true LMTs proved to be efficacious [23]. Moreover, a three-stent strategy was described, combining DK-Crush and Culotte technique, achieving excellent results [24].

Although more data and clinical trials are needed to develop proper evidence-based guidelines, three-stent implantation with Double-Trissing Crush should be taken into consideration as a viable strategy for LM trifurcation lesions in the proper set of patients.

Our case report presents a successful proof of concept for a three-stent strategy in a high SYNTAX score patient. At 6 months post-procedure, the patient’s evolution was favourable, without the occurrence of cardiovascular events, the control angiography revealing patent stents.

This technique also presents some limitations, such as the size of the vessels and the possibility of stent inflation from the proximal main branch into distal main branch, the risk of MB stent deformity, as well as the quantity of multiple layer metal at the carina level leading to a high risk of restenosis (maximum length of 4 mm, as measured with IVUS). The excessive stent metal risk of this technique must be evaluated. Intracoronary imaging thus becomes mandatory in this technique. This technique is time-consuming, while the number of resources used can become quite high. Another limitation may be the fact that the LAD and LCX must be considered as side branches due to the angulation with the left main. The middle vessel, that in the case of the LM is the RI, must be considered the main vessel. If we would consider the LAD as the main branch, crushing the side branch stents could cause problems in regard to struts coverage of the LCX ostium with multiple layers of stent struts making it very difficult to rewire the branch or lead to side branch compromise. We also consider, as limitations of the technique, the need for similar-sized branches and the difference in size between the proximal and distal main branch that influences the selection of the stent (the stent implanted according to the distal reference diameter of the main branch must be able to post expand to the proximal main vessel diameter).

## 4. Conclusions

The Double-Trissing Crush technique demonstrates potential as a viable strategy for left main trifurcation lesions even in high SYNTAX score patients. However, further research and clinical validation are necessary to standardize the technique, improve patient outcomes, and expand its applicability in broader patient populations.

## Data Availability

All data are available in the archive (data base) of the Clinical County Emergency Hospital of Oradea, Oradea, Bihor County, Romania.
